# Physiological Stratification of Patients With Angina Due to Coronary Microvascular Dysfunction

**DOI:** 10.1016/j.jacc.2020.03.051

**Published:** 2020-05-26

**Authors:** Haseeb Rahman, Ozan M. Demir, Faisal Khan, Matthew Ryan, Howard Ellis, Mark T. Mills, Amedeo Chiribiri, Andrew Webb, Divaka Perera

**Affiliations:** a British Heart Foundation Centre of Excellence and National Institute for Health Research Biomedical Research Centre at the School of Cardiovascular Medicine and Sciences, Kings College London, London, United Kingdom; b School of Biomedical Engineering and Imaging Sciences, King’s College London, London, United Kingdom

**Keywords:** coronary flow reserve, endothelial dysfunction, microvascular dysfunction, nitric oxide, stratified medicine, CBF, coronary blood flow, CFR, coronary flow reserve, CMD, coronary microvascular dysfunction, eNOS, endothelial nitric oxide synthase, FBF, forearm blood flow, hMR, hyperemic microvascular resistance, L-NMMA, *N*^G^-monomethyl-L-arginine, NOCAD, nonobstructive coronary artery disease, NOS, nitric oxide synthase

## Abstract

**Background:**

Coronary microvascular dysfunction (CMD) is defined by diminished flow reserve. Functional and structural CMD endotypes have recently been described, with normal and elevated minimal microvascular resistance, respectively.

**Objectives:**

This study determined the mechanism of altered resting and maximal flow in CMD endotypes.

**Methods:**

A total of 86 patients with angina but no obstructive coronary disease underwent coronary pressure and flow measurement during rest, exercise, and adenosine-mediated hyperemia and were classified as the reference group or as patients with CMD by a coronary flow reserve threshold of 2.5; functional or structural endotypes were distinguished by a hyperemic microvascular resistance threshold of 2.5 mm Hg/cm/s. Endothelial function was assessed by forearm blood flow (FBF) response to acetylcholine, and nitric oxide synthase (NOS) activity was defined as the inverse of FBF reserve to *N*^G^-monomethyl-L-arginine.

**Results:**

Of the 86 patients, 46 had CMD (28 functional, 18 structural), and 40 patients formed the reference group. Resting coronary blood flow (CBF) (24.6 ± 2.0 cm/s vs. 16.6 ± 3.9 cm/s vs. 15.1 ± 4.7 cm/s; p < 0.001) and NOS activity (2.27 ± 0.96 vs. 1.77 ± 0.59 vs. 1.30 ± 0.16; p < 0.001) were higher in the functional group compared with the structural CMD and reference groups, respectively. The structural group had lower acetylcholine FBF augmentation than the functional or reference group (2.1 ± 1.8 vs. 4.1 ± 1.7 vs. 4.5 ± 2.0; p < 0.001). On exercise, oxygen demand was highest (rate−pressure product: 22,157 ± 5,497 beats/min/mm Hg vs. 19,519 ± 4,653 beats/min/mm Hg vs. 17,530 ± 4,678 beats/min/mm Hg; p = 0.004), but peak CBF was lowest in patients with structural CMD compared with the functional and reference groups.

**Conclusions:**

Functional CMD is characterized by elevated resting flow that is linked to enhanced NOS activity. Patients with structural CMD have endothelial dysfunction, which leads to diminished peak CBF augmentation and increased demand during exercise. The value of pathophysiologically stratified therapy warrants investigation.

One-half of all patients with angina referred for angiography have nonobstructive coronary artery disease (NOCAD); those with coronary microvascular dysfunction (CMD) exhibit a poorer prognosis (1,2). CMD is diagnosed when there is diminished flow augmentation in response to a pharmacological vasodilator or reduced coronary flow reserve (CFR). A CFR <2.5 is associated with abnormal coronary flow with exercise and inducible ischemia on perfusion cardiac magnetic resonance imaging. We recently described 2 endotypes of CMD with distinct processes that contributed to low CFR: patients with functional CMD who have increased baseline flow (due to reduced microvascular resistance at rest); and patients with structural CMD who have reduced hyperemic flow (due to high minimal microvascular resistance) (3). Resting coronary blood flow (CBF) is regulated by nitric oxide synthase (NOS) and its role in the coronary circulation has been extrapolated from effects of vasoactive medication within the forearm circulation (4,5). In healthy patients, physical exercise involves synergistic adaptations between the peripheral and coronary vasculature to match CBF supply with myocardial oxygen demand, a process that is disrupted in structural CMD (3,6). The endothelium is responsible for translating mechanical forces (or shear stress) to smooth muscle dilatation in the coronary circulation and may have a similar role systemically during exercise (6). We sought to unravel the pathobiology of the 2 endotypes and hypothesized that: 1) elevated resting blood flow in functional CMD is a response to increased demand and is mediated by increased activity of the NOS pathway; and 2) structural CMD is reflective of a generalized disorder of vascular dilatation that affects peak flow in response to stress.

## Methods

Patients who underwent elective diagnostic angiography for investigation of exertional chest pain were enrolled in the study. Inclusion criteria were preserved left ventricular systolic function (ejection fraction >50%) and unobstructed coronary arteries (<30% diameter stenosis and/or fractional flow reserve >0.80). Exclusion criteria were intolerance to adenosine, chronic kidney disease (estimated glomerular filtration rate <30 ml/min/1.73 m^2^), concomitant valve disease (greater than mild on echocardiography), cardiomyopathy, or any neuromuscular comorbidity that could affect ability to perform bicycle exercise. Antianginal medications were stopped, and patients abstained from caffeine 24 h before all study visits. Antihypertensive medications were continued to ensure study participants were close to normotensive before study visits. The study protocol was approved by the UK National Research Ethics Service (17/LO/0203). The study was registered with the National Institute for Health Research UK Clinical Research Network portfolio database (Central Portfolio Management System identifier: 33170).

### Catheterization protocol

Catheterization was performed via the right radial artery using standard coronary catheters. All patients received 1 mg intravenous midazolam, 1 mg isosorbide dinitrate via the radial sheath, and intra-arterial unfractionated heparin (70 U/kg) before intracoronary physiological measurements. A dual pressure and Doppler sensor-tipped 0.014-inch intracoronary wire (Combowire, Volcano Philips, San Diego, California) was used to measure coronary pressure and flow velocity in the left anterior descending artery, as previously described (3,7). Hemodynamic measurements were recorded under resting conditions, during intravenous adenosine−mediated hyperemia (140 μg/kg/min) and continuously during bicycle exercise, using a specially adapted supine ergometer (Ergosana, Bitz, Germany) attached to the catheter laboratory table. Exercise began at a workload of 30 W and increased every 2 min by 20 W. When muscle weakness restricted increasing workloads, resistance was fixed at the maximum tolerated level, and exercise continued until exhaustion.

### Analysis of coronary physiological data

Signals were sampled at 200 Hz, with data exported into a custom-made study manager program (Academic Medical Centre, University of Amsterdam, Amsterdam, the Netherlands). Pan-cardiac cycle analysis and coronary wave intensity analysis were performed on custom-made software, Cardiac Waves (Kings College London, London, United Kingdom) as previously described (3,7). Microvascular resistance was calculated as the ratio of the distal mean coronary pressure and the average peak velocity. The supply/demand ratio was estimated as the average peak velocity/rate−pressure product (a measure of external stroke work) and denoted as supply/demand_EXT_. By wave intensity analysis, 4 dominant waves were identified and included in our analysis: 1) the backward compression wave, which causes flow deceleration during isovolumetric contraction in early systole; 2) the forward compression wave, which causes flow acceleration that is associated with peak aortic pressure; 3) the forward expansion wave, which causes flow deceleration associated with the fall in aortic pressure in late systole; and 4) the backward expansion wave, which causes flow acceleration during isovolumetric relaxation in early diastole. Total wave intensity was calculated as the sum of the area under the curve of the 4 principal waves, and coronary perfusion efficiency was calculated as the percentage of accelerating wave intensity in relation to total wave intensity.

Patients were classified into the reference group (CFR ≥2.5), functional CMD group (CFR <2.5; hyperemic microvascular resistance [hMR] <2.5 mm Hg/cm/s), or structural CMD group (CFR <2.5; hMR ≥2.5 mm Hg/cm/s) (3).

### Venous occlusion plethysmography

Forearm blood flow (FBF) was measured by venous occlusion plethysmography (Hokanson, Bellevue, Washington) as previously described (8). The right brachial artery was cannulated with a 27-gauge needle under local anesthetic cover with 1% lidocaine and saline vehicle or drugs infused at 1 ml/min. Saline was infused to establish a baseline before infusion of acetylcholine (Bausch & Lomb, Kingston-upon-Thames, United Kingdom) at 7.5 and 15 μg/min, adenosine (Sanofi, Guildford, United Kingdom) at 125 and 250 μg/min, and the nonselective NOS inhibitor, *N*^G^-monomethyl-L-arginine (L-NMMA) (Bachem, Rhein, Germany) at 2 and 4 μmol/min. Each vasoactive agent was infused at 2 doses for 6 min each, with FBF recorded over the last 3 min of each infusion (ml/min/100 ml forearm volume). The effect of each agent was calculated as the ratio of FBF compared with the preceding saline baseline. NOS activity was defined as the inverse of FBF reserve to L-NMMA.

### Cardiac biomarkers and mechanical function

N-terminal pro−brain natriuretic peptide levels were measured before angiography using conventional clinical assays. Diastolic function was assessed using standard transthoracic echocardiography measurements (9). Subjects also underwent high resolution cardiac magnetic resonance imaging with stress perfusion; these data were already presented in more detail (3). All scans were performed on a dedicated 3-T cardiac magnetic resonance scanner (Achieva TX, Phillips Healthcare, Best, the Netherlands). Contiguous short-axis slices were acquired from the base to the apex for calculation of left ventricular function and mass (CVI42, v5.1.1, Circle Cardiovascular Imaging, Calgary, Ontario, Canada).

### Statistical analyses

Sample size was estimated on the basis of the coprimary endpoints of FBF response to L-NMMA and acetylcholine. From previous reports, the expected FBF response in the reference group to L-NMMA and acetylcholine was 0.62 ± 0.03 and 4.1 ± 0.9, respectively (10). Assuming an allocation ratio among the reference group/functional/structural of 2:1:1, 56 patients would provide 95% power to detect a 10% difference in response to L-NMMA and 80% power to detect a 20% difference in response to acetylcholine. A total study sample of 85 was planned for powering both the exercise physiology study and FBF study, assuming 70% to 80% patients would undergo each study (3). Statistical analysis was performed using SPSS version 24 (IBM Corp., Armonk, New York). Normality of data was visually assessed (using histograms and the normal Q-Q plot) using the Shapiro-Wilk test. Continuous normal data are expressed as mean ± SD and compared using paired Student’s *t*-tests. Non-normal data are expressed as median (interquartile range [IQR]) and compared using the Mann-Whitney *U* test. Categorical variables are compared with chi-square tests. Repeated-measures analysis of variance of both drug doses was used to evaluate effects on FBF, whereas FBF in response to each agent was averaged out across low and high doses for the purposes of numerically displaying results among the study groups. A 2-tailed test for significance was performed for all analyses; p < 0.05 was considered statistically significant. The p values and 95% confidence intervals presented in this study were not adjusted for multiplicity; therefore, inferences drawn from these statistics might not be reproducible.

## Results

**Figure 1:**
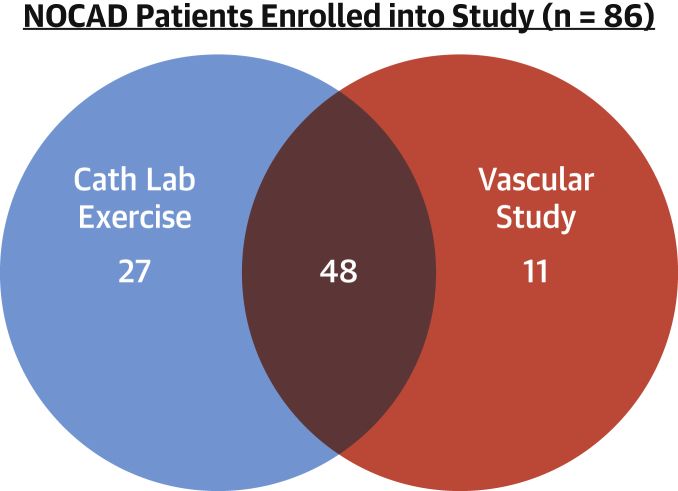
Final Studies Undertaken All patients with angina and no obstructive coronary disease (NOCAD) enrolled in the study had assessment of coronary flow reserve (n = 86). Cath Lab Exercise = invasive catheter laboratory exercise physiology; Vascular Study = forearm venous occlusion plethysmography.

A total of 86 patients with angina and NOCAD underwent coronary microvascular function testing; they were enrolled into the study and subsequently classified into the reference group (n = 40), the functional CMD (n = 28) group, and the structural CMD (n = 18) group using the preceding pre-specified definition. Seventy-five patients underwent catheter laboratory exercise, and 59 patients underwent peripheral vascular function testing (Figure 1). Patients with structural CMD had higher incidences of hypertension and diabetes compared with patients in the functional CMD and reference groups (78% vs. 43% vs. 63%; p = 0.03; and 44% vs. 18% vs. 23%; p = 0.04). Patients with structural CMD were taking a greater number of antihypertensive agents (1.00 ± 1.00 vs. 0.46 ± 0.74; p = 0.046) but a similar number of anti-anginal medications. N-terminal pro−brain natriuretic peptide values were highest in patients with structural CMD compared with those with functional CMD and reference group patients (132 pg/ml [IQR: 82 to 179 pg/ml] vs. 69 pg/ml [IQR: 32 to 116 pg/ml] vs. 34 pg/ml [IQR: 22 to 90 pg/ml]). Overall diastolic function and exercise time and external work were no different between both CMD endotypes (Table 1).

**Table 1:** Patient Characteristics

	Reference Group (n = 40)	Functional CMD (n = 28)	Structural CMD (n = 18)	p Value
Clinical characteristics				
Age, yrs	57 ± 10	57 ± 12	57 ± 9	0.99
Female	27 (68)	25 (89)	15 (83)	0.51
Hypertension	25 (63)	12 (43)	14 (78)	0.03
Diabetes	9 (23)	5 (18)	8 (44)	0.04
No. of antianginal Rx	0.88 ± 0.85	0.89 ± 0.92	0.59 ± 0.51	0.31
No. of antihypertensive Rx	0.50 ± 0.60	0.46 ± 0.74	1.00 ± 1.00	0.05
Cardiac biomarkers and function				
NT-proBNP, pg/ml	34 (22–90)	69 (32–116)	132 (82–179)	0.01
Echo, E/é	6.9 ± 2.2	7.5 ± 3.0	8.4 ± 2.3	0.39
Indexed LV mass, g	44.6 ± 16.9	42.9 ± 12.1	42.8 ± 10.4	0.99
LVEF, %	64.8 ± 5.3	67.4 ± 6.8	66.0 ± 4.0	0.53
Exercise performance				
Exercise time, s	417 ± 146	412 ± 117	364 ± 93	0.21
Exercise work, W	72 ± 28	65 ± 21	61 ± 33	0.65

Values are mean ± SD, n (%), or median (interquartile range). Risk factors include those with an established diagnosis who required treatment with prescription therapy (Rx). p value refers to the difference between functional and structural microvascular dysfunction (coronary microvascular dysfunction [CMD]) groups.

LV = left ventricular; LVEF = left ventricular ejection fraction; NT-proBNP = N-terminal pro–brain natriuretic peptide; Rx = prescriptions.

### Resting physiology

Resting CBF was higher among patients with functional CMD compared with patients with structural CMD and reference group patients (24.6 ± 5.7 cm/s vs. 16.6 ± 3.9 cm/s vs. 15.2 ± 4.7 cm/s; p < 0.001 for both). Resting myocardial blood flow on cardiac magnetic resonance imaging had a similar pattern among the groups (1.42 ± 0.40 ml/min/g vs. 1.23 ± 0.27 ml/min/g vs. 1.13 ± 0.21 ml/min/g; p = 0.21 vs. structural and p = 0.003 vs. reference group patients). Patients with functional and structural CMD had higher total resting wave energy than reference group patients (1.71 ± 1.2 Wcm^−2^s^−2^ vs. 1.46 ± 0.6 Wcm^−2^s^−2^ vs. 0.9 ± 0.8 Wcm^−2^s^−2^; p = 0.01 and 0.001). Despite this, overall coronary perfusion efficacy was similar between the groups (59 ± 12% vs. 60 ± 12% vs. 63 ± 11%; p = 0.69 and p = 0.22).

Supply/demand_EXT_ was higher in patients with functional CMD than those with structural CMD and in reference group patients (13.5 ± 3.2 cm/mm Hg × 10^−2^ vs. 8.2 ± 2.0 cm/mm Hg × 10^−2^ vs. 9.1 ± 3.4 cm/mm Hg × 10^−2^; both p < 0.001).

**Figure 2:**
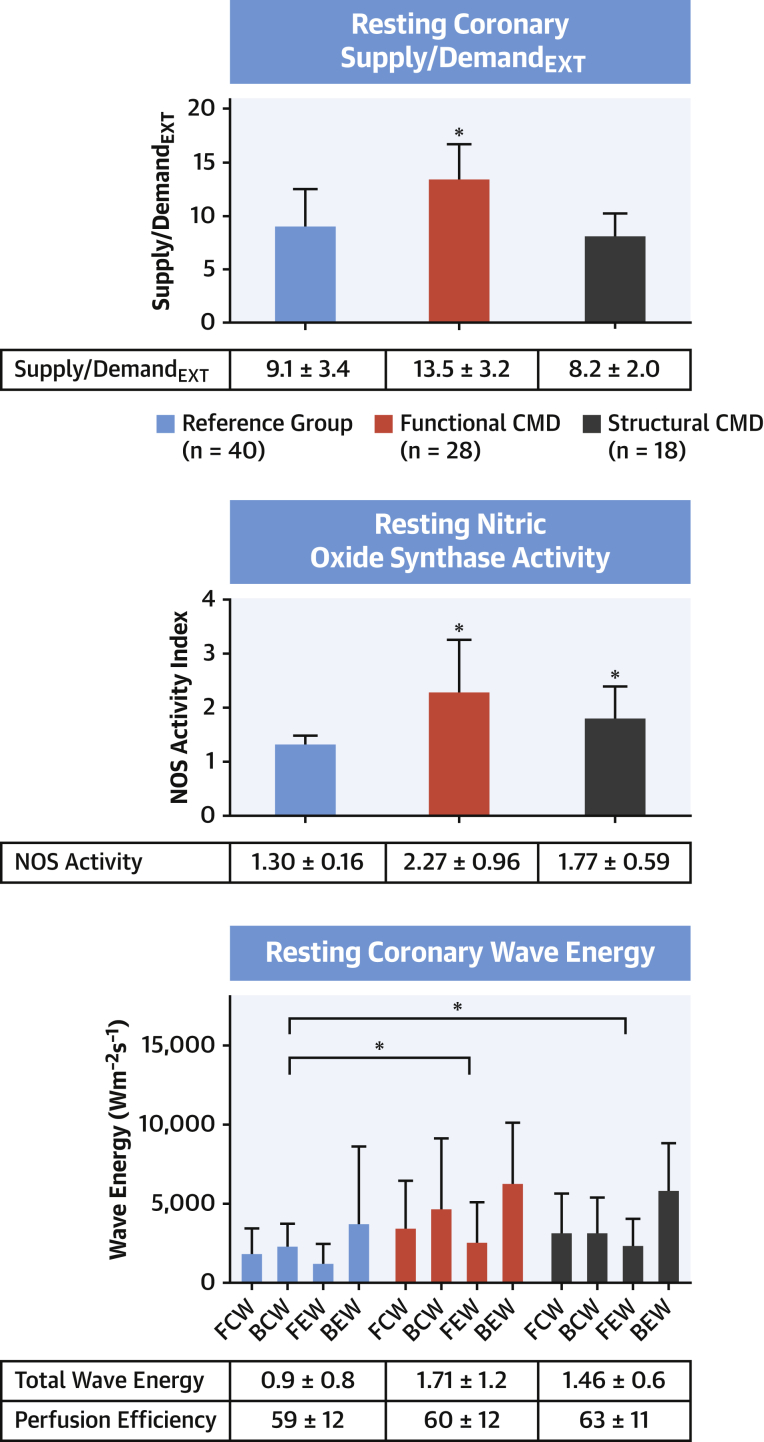
Resting Physiological Abnormalities in CMD **(Top)** Patients with functional coronary microvascular dysfunction (CMD) have higher supply/demand_EXT_ (average peak velocity/rate−pressure product, in cm/mm Hg × 10^−2^) than structural CMD and reference group patients. **(Middle)** Both functional and structural CMD have elevated nitric oxide synthase (NOS) activity calculated as resting forearm blood flow/forearm blood flow during infusion of *N*^G^-monomethyl-L-arginine. **(Bottom)** Both functional and structural CMD have increased resting wave energy calculated using wave intensity analysis. ∗Statistically significant different from reference group, where p < 0.05. Numbers depict mean values and **error bars** depict SD. BCW = backward compression wave; BEW = backward expansion wave; FCW = forward compression wave; FEW = forward expansion wave.

The reduction in FBF in response to L-NMMA was greatest in patients with functional CMD compared with those with structural CMD and reference group patients (flow reserve: 0.49 ± 0.13 vs. 0.61 ± 0.14 vs. 0.79 ± 0.09; p = 0.01). NOS activity was highest in patients with functional CMD compared with those with structural CMD and reference group patients (2.27 ± 0.96 vs. 1.77 ± 0.59 vs. 1.30 ± 0.16; p < 0.001 both). The determinants and patterns of resting physiology are summarized in Figure 2.

### Response to exercise and vasoactive agents

**Figure 3:**
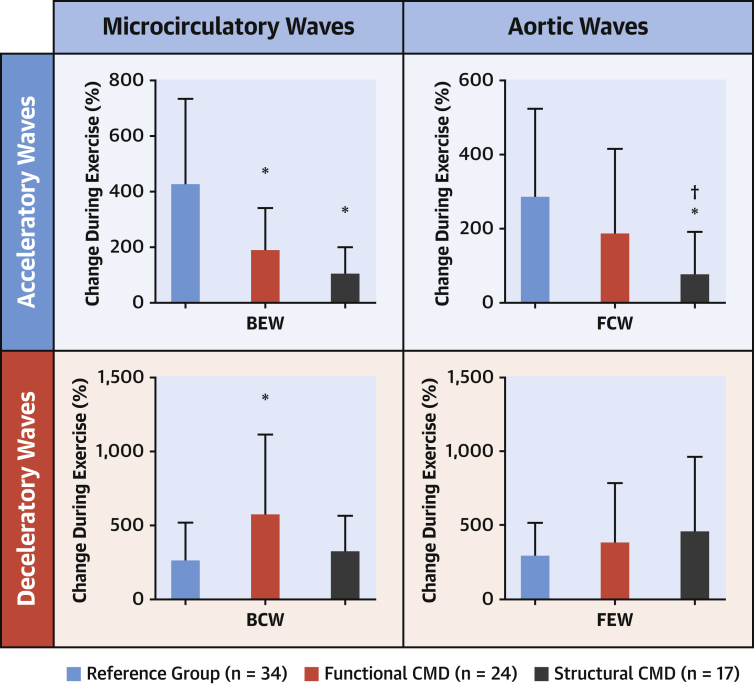
Changes in Coronary Wave Energies During Exercise in Reference Group Patients and in Patients With Functional and Structural CMD ∗Significant difference from reference group. †Significant difference between CMD endotypes, both p < 0.05. **Numbers** depict mean values and **error bars** depict SD. Abbreviations as in Figure 2.

Although functional and structural CMD had similar CFRs (1.98 ± 0.33 vs. 1.96 ± 0.38; p = 0.82), the former had abnormally high resting flow and the latter had diminished peak flow. By definition, coronary microvascular resistance during peak exercise was higher in patients with structural CMD compared with those with functional CMD and reference group patients (3.1 ± 1.8 mm Hg/cm/s vs. 1.9 ± 0.4 mm Hg/cm/s vs. 2.1 ± 0.5 mm Hg/cm/s; p < 0.001 structural compared with both). CBF during peak exercise was lower in patients with structural CMD compared with those with functional CMD (25 ± 5 cm/s vs. 34 ± 11 cm/s; p < 0.001) and similar to that in reference group patients (27 ± 8 cm/s; p = 0.41). During exercise, the magnitude of all waves increased in all subjects, but accelerating wave energy increased preferentially in reference group patients, whereas decelerating energy increased in both CMD groups. Perfusion efficiency was lower in both patients with structural CMD and functional CMD compared with patients in the reference group (41 ± 10% vs. 46 ± 9% vs. 65 ± 15%) (Figure 3).

**Figure 4:**
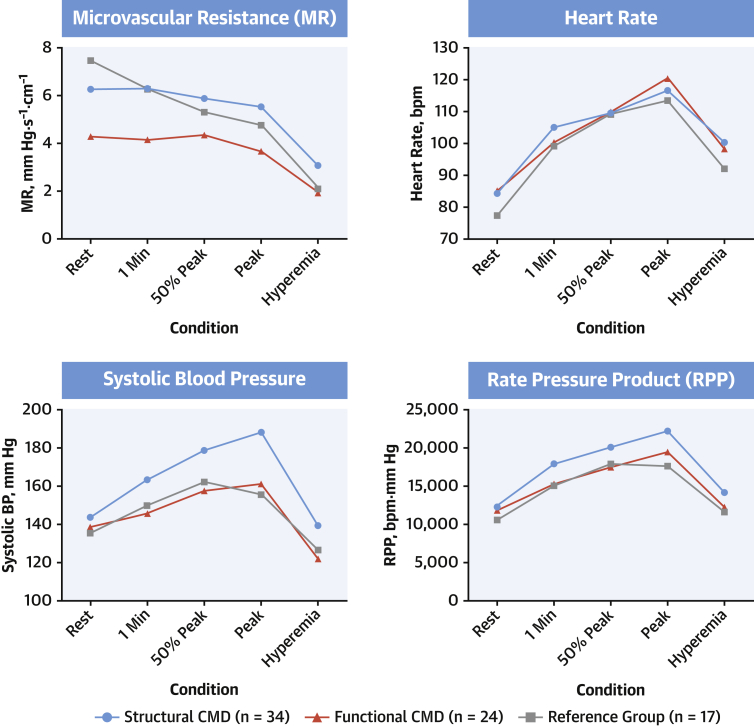
Systemic Hemodynamic and Coronary Circulation Response to Stress Patients with structural CMD had varying hemodynamic responses to supine bicycle exercise and adenosine-induced hyperemia compared with those with functional CMD and those in the reference group. **Numbers** depict mean values. 1 min = after 1 min of exercise; 50% = 50% of maximal exercise time; hyperemia = adenosine-induced hyperemia; MR = microvascular resistance; peak = immediately before exercise was discontinued due to exhaustion; RPP = rate−pressure product.

In terms of systemic hemodynamics, during peak exercise, the structural group had higher systolic blood pressure (188 ± 25 mm Hg) than the functional (161 ± 27 mm Hg; p = 0.004) and reference group patients (156 ± 30 mm Hg; p < 0.001), and consequently, had a higher rate−pressure product (22,157 ± 5,497 beats/min/mm Hg vs. 19,519 ± 4,653 beats/min/mm Hg vs. 17,530 ± 4,678 beats/min/mm Hg; p = 0.12 vs. the functional group and p = 0.004 vs. the reference group) (Figure 4).

**Figure 5:**
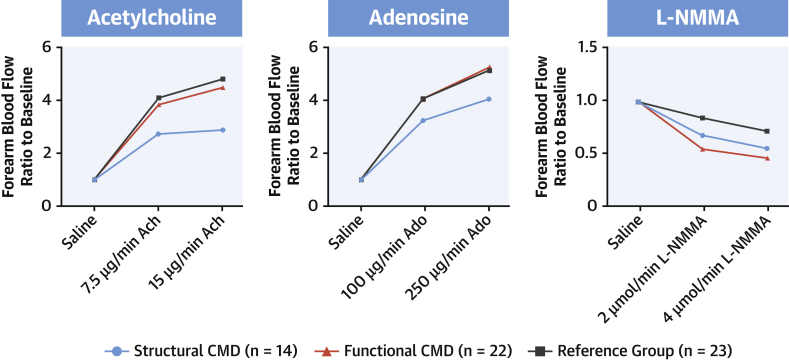
Results From Venous Occlusion Plethysmography Both functional and structural CMD demonstrate greater vasoconstriction in response to *N*^G^-monomethyl-L-arginine (L-NMMA), whereas structural CMD also demonstrated reduced vasodilatation to acetylcholine (ACh) and adenosine (Ado). **Numbers** depict mean values.

The response to vasoactive agents was also significantly different across the groups. The increase in FBF in response to acetylcholine was diminished in the structural group compared with the functional and reference group patients (2.1 ± 1.8 vs. 4.1 ± 1.7 vs. 4.5 ± 2.0; p = 0.001 vs. the functional group and p = 0.0006 vs. the reference group) and was diminished in response to adenosine (3.8 ± 1.8 vs. 4.8 ± 2.8 vs. 4.7 ± 1.7; p = 0.04 vs. the functional group and p = 0.03 vs. the reference group) (Figure 5). There were no differences in FBF vasodilatory response to acetylcholine or adenosine between reference group patients and patients with functional CMD (p = 0.46 and p = 0.90, respectively). These results are summarized in the Central Illustration.

**Central Illustration:**
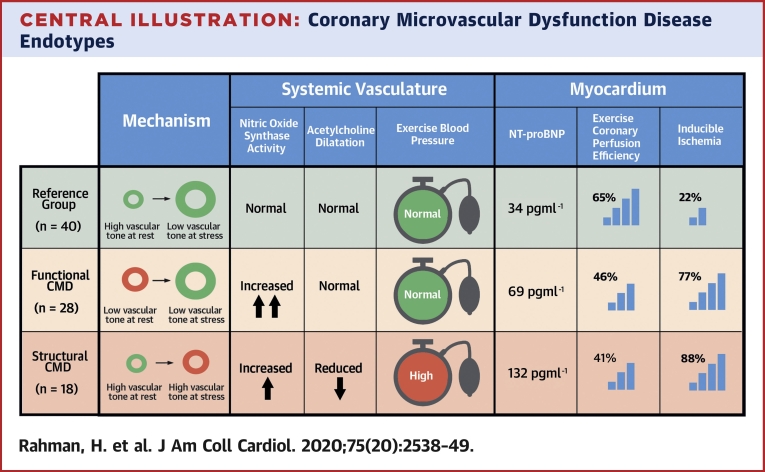
Coronary Microvascular Dysfunction Disease Endotypes Summary of distinct coronary, myocardium, and systemic changes associated with each disease endotype compared with the reference group of patients (3). CMD = coronary microvascular dysfunction; NT-proBNP = N-terminal pro–brain natriuretic peptide.

## Discussion

This study showed that functional and structural CMD endotypes have inefficient cardiac-coronary coupling during exercise compared with reference group patients with preserved CFR. This was congruent with our previous demonstration of higher rates of inducible myocardial ischemia during vasodilator stress (3). Although both CMD endotypes display generalized disorders of the circulation, the underlying pathophysiological determinants are distinct. In functional CMD, there is increased myocardial perfusion at rest, which is partly explained by increased external cardiac work but may also be due to greater potential energy at rest, which may, in turn, be explained by the higher wave energy at rest. Increased perfusion appears to be mediated by enhanced NOS activity, which leads to submaximal coronary vasodilatation at rest, which, in turn, reduces the vasodilator reserve in response to stress. Structural CMD had similar resting microvascular resistance to reference group subjects at rest but had markedly elevated microvascular resistance during both exercise and vasodilator stress, which led to maladaptive exercise physiology and global myocardial ischemia, respectively. In addition, the structural group had impaired peripheral endothelium-dependent dilatation and exaggerated exercise hypertension, which caused increased afterload and increased myocardial oxygen demand, which exacerbated the supply deficit. The resting pathophysiology in both endotypes of CMD might benefit from metabolic modulation, whereas structural CMD might also derive benefit from therapies designed to reduce afterload and myocardial oxygen demand during effort.

### Structural CMD

The historical perception of CMD was that it was related to high vascular resistance during increased demand or vasodilatation. Interestingly, in our study cohort, the patients who conformed to this pathophysiological subtype (whom we classified as having structural CMD) represented less than one-half of all patients with impaired CFR. The structural CMD group had more established cardiovascular risk factors and poorly controlled hypertension, a process similarly associated with diminished maximal flow (11). Exercise-induced hypertension led to increased myocardial oxygen demand during exercise within this group. Attenuated reduction in afterload with exercise interrupted the usual synergistic response of the coronary and peripheral circulations and predispose patients to ischemia (6). Systemic vasodilatation in response to acetylcholine, which in health generates endogenous nitric oxide via endothelial nitric oxide synthase (eNOS), was impaired in patients with structural CMD. Therefore, shear stress during exercise might not translate to appropriate eNOS-mediated dilatation of coronary and peripheral vascular smooth muscle, which could lead to enhanced vascular tone in both beds. Shear stress−mediated increases in resting CBF are believed to be protective, and it has been suggested this mechanism might be defective in patients with diabetes, which could involve the eNOS pathway. Genetic polymorphisms involving eNOS dysfunction have been implicated in CMD; whether genotyping individuals might have a role in classifying the disease state and in guiding therapy is a further exciting future prospect (12).

Patients with NOCAD with elevated hyperemic resistance have a higher incidence of refractory angina. Our study similarly identified a numerical trend toward a higher burden of inducible myocardial ischemia and biomarker changes that suggested that structural CMD was a more advance disease endotype (3,13). The impaired augmentation of the diastolic acceleratory wave suggested impaired lusitropy, whereas elevated N-terminal pro−brain natriuretic peptide levels suggested an early diastolic dysfunction state (14). Whether elevated resting flow in functional CMD precede changes that lead to structural CMD needs to be assessed with longitudinal studies. A similar bimodal pattern of impaired flow reserve was observed in the diabetic coronary circulation (15). Studies that dichotomized patients with CMD based upon maximal flow demonstrated that mortality rate was higher in structural CMD, whereas major adverse cardiovascular events were higher in functional CMD compared with those with isolated endothelial dysfunction, which supported this notion of disease progression identified by measuring minimal microvascular resistance (16). The state of raised minimal microvascular resistance in structural CMD might be surmountable over time with appropriate treatment; therefore, the term structural should not be considered to imply an irreversible disease process. In the first instance, judicious blood pressure control using large vessel vasodilators could enhance the normal synergistic adaptation to exercise observed in health in structural CMD. Endothelial function modulation, including pleiotropic actions of statins, might theoretically exert a greater effect on structural CMD compared with functional CMD classes and need to be studied in a placebo randomized study.

### Functional CMD

Although defined by demonstrating normal minimal microvascular resistance, the pathophysiological hallmark of functional CMD is increased resting flow. This was previously described and was associated with greater risk of major adverse cardiovascular events (17). Coronary autoregulation is the intrinsic capacity of microcirculation to maintain distal flow in response to a range of physiological perfusion pressures, and elevated flow can represent either disordered autoregulation or appropriate autoregulation in the face of increased myocardial oxygen demand. Our study provided indirect evidence in favor of the latter and also suggested that elevated resting coronary flow might be mediated by elevation of NOS activity. Alternatively, it was possible that increased nitric oxide activity was an adaptation to abnormally increased vasoconstrictor responsivity. For instance, patients with microvascular angina were shown to exhibit a greater contractile response to endothelin-1 (18). A study of patients with the historical cardiac syndrome X definition demonstrated accentuated responsivity following in vivo administration of endothelin-A antagonism, and modulation of this pathway is currently being studied as a novel therapeutic target in CMD (10,19). Although we measured the effect of NOS inhibition in the peripheral vascular bed, good correlation between the coronary vascular bed was previously demonstrated (4). Nitric oxide dilatation of proximal arteries occurs preferentially to preserve vasodilator reserve in the distal arterioles; therefore, this may serve as an initially protective mechanism to maintain metabolic vasodilatory reserve (20,21). Although external myocardial work in the form of rate−pressure product is not elevated in functional CMD, higher basal metabolic requirements can underlie elevated resting CBF (22). Higher resting wave energy, but no net increment in perfusion efficiency implies an inefficient resting state more pronounced in functional than structural CMD. An inefficient metabolic state may represent the “common soil” of CMD and heart failure with preserved ejection fraction (23). In patients with diabetes, depressed myocardial energetics precedes any overt changes in microvascular function; similarly, diastolic dysfunction occurs late within the disease natural history (24,25). In heart failure with preserved ejection fraction, reduced CFR is attributable to both elevated resting CBF and diminished hyperemic CBF, resembling both functional and structural CMD endotypes; emerging data increasingly suggest that CMD may be a pathological precursor of heart failure with preserved ejection fraction (26,27).

### Personalized therapy

Numerous pharmacological and non-pharmacological therapies have been studied in patients with chest pain and NOCAD. However, previous studies have been limited by enrolling small numbers of participants with variable inclusion criteria, using differing diagnostic test thresholds and measuring dissimilar endpoints (28,29). With an increased armamentarium of novel therapies, such as immunomodulation for treatment of atherosclerotic coronary artery disease, there is a movement toward stratifying populations to determine who will derive the greatest benefit (30). The migration from empirically treating NOCAD to identifying CMD has already demonstrated superiority, and it is likely the heterogeneous population of patients with CMD can be further refined (31). Measuring resistance is relatively easy to do, and the resistance indexes (either Doppler-based hMR or the thermodilution-based index of microvascular resistance) are automatically calculated when determining CFR using currently available pressure sensor-tipped guide wires such as the Combowire (Volcano Philips) or Pressure Wire X (Abbott, Chicago, Illinois) (32,33). In combination with CFR, hMR or index of microvascular resistance measurement would distinguish the 2 distinct CMD endotypes with different dysfunctional pathways leading to exercise ischemia. Identifying patients with CMD and coronary vasospasm has already been a huge step forward in terms of personalizing therapy for NOCAD (31,34). CorMicA investigators have similarly stratified patients based upon CFR, index of microvascular resistance, and vasospastic abnormalities, demonstrating differences in peripheral endothelin-1 function among endotypes, paving the way for repurposing of targeted pharmacotherapy (18). Placebo-controlled trials will be the next step to identifying if targeted therapy based upon CMD endotypes will yield superior outcomes to empirical treatment of all-comers with diminished CFR. Understanding the mechanistic basis of CMD endotypes may guide the development of novel therapies and improve our management of this common yet poorly treated condition.

### Study limitations

This was a mechanistic single-center study with relatively small numbers of patients in each group. Our study design did not distinguish whether nNOS or eNOS or both were upregulated in both CMD endotypes and whether the findings in the forearm truly mimicked those within the coronary circulation. This was an observational study, and it was unclear if this classification could guide therapeutics to sufficiently improve patient outcome. This will need to be assessed in a placebo randomized trial. Invasive CFR is currently the most accepted method for classifying CMD in patients with NOCAD. Like all biological measurements, CFR is a continuous variable, and, for this study, a dichotomous CFR threshold of 2.5 was adopted to define CMD, acknowledging that a lower threshold might have had enhanced specificity at the cost of sensitivity. Moreover, because our reference group were not healthy volunteers but had symptoms that led to angiography, adopting a 2.5 threshold ensured that their endothelium-independent microvascular function was truly normal. Patients in the reference group might have had occult coronary abnormalities, such as endothelial dysfunction or coronary vasospasm, that could be unmasked during provocation testing. During cardiac catheterization, pre-medication using radial nitrates were necessary to enable bike exercise via this protocol. Although the same dose was administered to each study participant, this might have affected the coronary physiology results. However, with most angiography being performed radially, this method was more representative of contemporary practice.

## Conclusions

CMD is associated with systemic vascular endothelial abnormalities. Functional and structural endotypes, defined by reduced resting and high minimal microvascular resistance, respectively, have different mechanisms of ischemia during exercise. In functional CMD, inefficient cardiac-coronary coupling during peak exercise and during rest leads to higher myocardial oxygen demand in the face of submaximally exhausted vasodilatory reserve at rest. In structural CMD, systemic endothelium dilatory function leads to systemic hypertension and increased myocardial work, whereas coronary blood flow augmentation is diminished. This is further compounded by an inefficient cardiac-coronary coupling state.

Perspectives **COMPETENCY IN MEDICAL KNOWLEDGE:** Measurement of microvascular resistance at rest and during pharmacologically induced hyperemia identifies 2 types of coronary microvascular dysfunction. In one endotype, CBF is impaired at rest due to nitric oxide dysregulation, whereas in the other, endothelial dysfunction limits peak hyperemic blood flow.

**TRANSLATIONAL OUTLOOK:** Therapeutic studies should stratify patients with microvascular dysfunction based on impairment of resting or hyperemic flow.
